# Effects of ball milling treatment on physicochemical properties and digestibility of Pacific oyster (*Crassostrea gigas*) protein powder

**DOI:** 10.1002/fsn3.705

**Published:** 2018-07-16

**Authors:** Zhenyu Wang, Sijia Chang, Yajing Li, Liu Kong, Di Wu, Lei Qin, Cuiping Yu, Chao Wu, Ming Du

**Affiliations:** ^1^ National Engineering Research Center of Seafood School of Food Science and Technology Dalian Polytechnic University Dalian 116034 China

**Keywords:** ball milling, *Crassostrea gigas*, *in vitro* digestibility, physicochemical properties

## Abstract

The oyster protein was ball milling treated in this work, and the effects on particle size, conformation, physicochemical properties, and *in vitro* protein digestibility (IVPD) were investigated. After ball milling treatment, the particle size obviously decreased, and the protein powder became denser and more homogeneous. The ball milling treatment could not change the primary structure of oyster protein. However, it could affect the secondary structure and physicochemical properties. The disulfide bond increased from 8.18 to 9.14 μmol/g protein, while the protein surface hydrophobicity index increased from 0.088 to 0.176. The decreasing water‐holding capacity from 390% to 226% and the increasing oil‐binding capacity from 91.2% to 189.1% were related to the alterations of conformation and physicochemical properties. Ball milling could also improve the IVPD from 54.6% to 82.4%. These results provided theoretical basis for the application of ball milling treatment in the utilization of oyster protein in the food industry.

## 1 INTRODUCTION

Oyster, one of the largest cultured shellfish in the world, is popular in different countries and regions (Li, Yu, & Yu, 2006). Oyster, well known as an excellent source of protein and minerals for human diet nutrition, is particularly rich in essential amino acids, iron, zinc, and copper, as well as unsaturated fatty acids like eicosapentaenoic acid and docosahexaenoic acid (Shen & Su, 2017). Nutrients in oyster are well consistent with Recommended Dietary Allowances (RDAs) in people's content (Otten, Hellwig, & Meyers, 2006). In particular, with high quality of its protein nutrition among aquatic products, the oyster protein is also known as “marine milk,” which can be used as an important raw material of protein powder.

Protein powder, as an important raw material in food processing, has a wide range of uses and development prospects. It can be used as a nutritional supplement to provide essential nutrition for children, old people, athletes, preoperative or postoperative patients and weight loss groups. Currently, this kind of nutritional protein powder is mainly made of soy protein isolate or whey protein (Bauer et al., 2015; Lammert, Olabi, Kalache, Brooks, & Tong, 2014). Considering the amino acid composition and the nutrition of oyster protein, it is feasible using oyster protein as the raw material in the nutritional protein powder (Linehan, O'connor, & Burnell, 1999). On the other hand, oyster protein powder can be also used as a raw material in some favoring and sauces, due to its special flavor (Je, Park, Jung, & Kim, 2005; Kingsley et al., 2015). Thus, smaller particle size and better physicochemical properties are needed during its utilization. The process of pulverization plays an important role changing physicochemical properties and reducing particle size of oyster protein powder.

Superfine grinding technology, which crushes solid materials into powders less than 25 μm in diameter, has shown potential for production of nutraceuticals and functional foods (Chen, Weiss, & Shahidi, 2006; Wuhan, Erqi, & Ke, 2015). And ball milling treatment is a typical representative amongst the superfine grinding methods used in starches (Anzai, Hagiwara, Watanabe, Komiyama, & Suzuki, 2011; Dhital, Shrestha, & Gidley, 2010; Martinez‐Bustos, Lopezsoto, San, Zazuetamorales, & Velezmedina, 2007; Tan et al., 2015), soybean protein isolate (Liu et al., 2017), chocolates (Alamprese, Datei, & Semeraro, 2007; Toker et al., 2017), mushroom powder (Wang et al., 2016), and wheat flours (Thanatuksorn, Kawai, Kajiwara, & Suzuki, 2009). As an eco‐friendly and high‐efficiency technology, ball milling changes the molecular arrangement of the surface of the food material, the individual particles for the human tongue to distinguish, and the crystal structure and the arrangement of electrons, resulting in several special effects, such as small size effect of protein particles, which are not available in the original food material. Ball‐milled products have shown significantly changes in physical and chemical properties; however, few reports is available about the effect of the ball milling on the oyster protein and more works need to do to reveal the changes on the functional properties and digestibility of ball‐milled oyster protein (BMOP).

In this study, oyster protein was used as the raw material and ball milling method was carried out to investigate the effect of ball milling on the physicochemical properties, such as particle size, water‐holding capacity (WHC), oil‐binding capacity (OBC), surface hydrophobicity index, disulfide linkage group contents, and circular dichroism. Effects on *in vitro* digestibility and free amino acid contents were also studied. This work aimed to provide theoretical basis for the utilization and development of oyster protein.

## 2 MATERIAL AND METHODS

### 2.1 Materials

The fresh oyster (*Crassostrea gigas*) was bought from Meilin Market (Ganjingzi District, Dalian, China) and transferred to the lab immediately with ice bath. The shells of oyster were shucked, and the tissue was collected and freeze to −80°C for further protein extraction. The crude protein concentration on a wet basis was 7.50±0.50% in the oyster tissue determined by the Kjeldahl method. The oyster tissue was fully dispersed in deionized water (1:3, w/w) by an IKA disperser (IKA^®^ Works, Inc., Germany) at 12,000 rpm for 3 min. The soliquoid was stirred using a magnetic stirrer (Corning) for 40 min at pH 11.0 under 45°C. Then, the mixture was centrifuged at 12,000 × *g* for 15 min at 4°C. The supernatant was adjusted to pH 5.0 with 6 M HCl and maintained for 1 hr. The oyster protein was obtained as precipitate by centrifugation at 12,000 × *g* under 4°C for 15 min. After freeze‐dried and defatted using cold acetone (1:10, w/v), the oyster protein was finally obtained with a protein content of 82.0%.

### 2.2 Ball milling treatment of oyster protein

A ball milling equipment (Mixer Mill MM400; Retsch Technology, Haan, Germany) was used to produce BMOP according to a previous report (Yu et al., 2018). Briefly, two grinding tanks (volume of 50 ml) were filled with 5 g oyster protein, respectively, along with a stainless steel ball (25 mm Ø) in each tank. The oyster protein was ball‐milled into powder at a program at 20 Hz for 4, 8, 12, 16, 20 min, while a sample treated for 2 s was set as the control point of 0 min. The BMOP samples were then transferred into an airtight dry petri dish and further analyzed within 2 days.

### 2.3 Scanning electron microscopy (SEM)

The morphology of BMOP particles was observed using a scanning electron microscopy (JSM‐6390LV; Tokyo, Japan) at an accelerating voltage of 2.0 kV. For each sample, about 0.01 g BMOP was attached onto a copper sheet with a piece of double‐faced adhesive tape and sputter‐coated with gold. The image was obtained at 500× magnifications.

### 2.4 Particle size determination

The BMOP samples (0.5 g) were dispersed in 10 ml phosphate buffer (0.05 M, pH = 8.0) and fully mixed by a shaking table for one hour at 150 rpm. After centrifugation at 4,500*×g* for 5 min, the particle size distribution was investigated using a Zetasizer 3000 HSA (Malvern, Worcestershire, UK). The refractive index and absorption parameter were set as 1.330 and 0.001, respectively.

### 2.5 Determination of water‐holding capacity and oil‐binding capacity

Water‐holding capacity and oil‐binding capacity (OBC) were determined according to a previous report (Beuchat, 1977). Briefly, 0.1 g of BMOP samples was mixed with distilled water or soybean oil (1:50, w/v). The mixture was vortexed for 1 min and maintained for 1 hr and then centrifuged at 4,500*×g* for 15 min. After centrifugation, the supernatant was completely decanted. WHC (%) and OBC (%) were calculated as follows:

$$\text{WHC}\left(\%\right)=\frac{W_{T}-W_{R}}{W_{T}}\times 100\%$$

where *W*_*R*_ means mass of water released from BMOP samples after centrifugation, and *W*_*T*_ means total mass of water in BMOP samples before centrifugation.

$$\text{OBC}\left(\%\right)=\frac{O_{T}-O_{R}}{O_{T}}\times 100\%$$

where *O*_*R*_ means mass of oil released from BMOP samples after centrifugation, and *O*_*T*_ means total mass of oil in BMOP samples before centrifugation.

### 2.6 Sodium dodecyl sulfate‐polyacrylamide gel electrophoresis

Sodium dodecyl sulfate‐polyacrylamide gel electrophoresis (SDS‐PAGE) was used to observe the protein distribution of different BMOP samples according to the reported method (Chicón, Belloque, Alonso, & López‐Fandiño, 2009) with slight modification. The protein distribution was identified using premixed protein marker (Takara Bio Inc., Japan). The BMOP samples were mixed with the sample buffer (10%, w/v) and shaken for 12 hr. The samples and markers were loaded on hand‐cast 5% stacking gel (pH 6.8) and 10% separating gel (pH 8.8) followed by electrophoresis using a Mini‐Protein II electrophoresis system (Bio‐Rad Laboratories, Hercules, CA, USA) at 15 mA, which increased to 30 mA when the sample bands reached the separation gel. Then, the gel was stained with Coomassie Blue staining (0.1% Coomassie Brilliant Blue R‐250, 25% isopropyl alcohol, 10% acetic acid) for an hour, and then decolored with decoloring liquid (10% acetic acid, 5% ethanol) for 2 hr. All images were analyzed by Quantity One software version 4.6.2.70 (Bio‐Rad Laboratories) based on the separation of MW standards.

### 2.7 Circular dichroism spectropolarimetry

On the basis of the previous report with slightly modification (Jiang et al., 2014), circular dichroism (CD) spectropolarimetry was scanned at the far‐UV range (260–180 nm) in order to reveal the secondary structure of BMOP. BMOP (40 mg/ml) were centrifuged at 8,000 × *g* for 15 min at 25°C. The supernatants (3 ml) were used for circular dichroism spectropolarimetry measurements (CD‐J1500; Jasco International, Tokyo, Japan). Secondary structures including α‐helix, β‐sheet, β‐turn, and random coil were calculated using Spectra Manager Software (Jasco International).

### 2.8 Sulfhydryl and disulfide linkage group contents

Total sulfhydryl (SH_T_) and free sulfhydryl (SH_F_) was determined according to the method of Shimada and Cheftel (Shimada & Cheftel, 1988) using DTNB reagent modified by Sun, Liu, et al. (2015). The absorbance at 412 nm (A_412_) was detected by a UV‐VIS spectrophotometer (UV 2400, SOPTOP, Shanghai, China). The contents of SH_T_, SH_F_, and the disulfide linkage group (SS) were calculated as follows:

$$SH_{T}\left(\mu mol/g\;prot\right)=\frac{73.53\times A_{412}\times D}{C}$$

$$SH_{F}\left(\mu mol/g\;prot\right)=\frac{73.53\times A_{412}\times D}{C}$$

$$SS\left(\mu mol/g\;prot\right)=\frac{SH_{T}-SH_{F}}{2}$$

where D is the dilution factor = 3.02, and C is the protein content in BMOPP samples detected by Kjeldahl method.

### 2.9 Determination of surface hydrophobicity index

The protein surface hydrophobicity index (PSH) of BMOP was determined using the method of hydrophobic chromophore bromophenol blue (BPB) according to Chelh's report (Chelh, Gatellier, & Santé‐Lhoutellier, 2006) with slight modification. Briefly, 5 mg of BMOP sample was dispersed in deionized water (1:1,000, w/v) and then vortexed thoroughly with 500 μl BPB reagent (1 mg/ml) in a 10 ml centrifuge tube for 10 min. The supernatant was obtained by centrifugation at 6,000 × *g* for 10 min. The absorbance of the supernatant was detected at 595 nm using a UV‐VIS spectrophotometer (UV2400; Shanghai Sunny Hengping Instrument Co. Ltd., China), while a same volume of deionized water was set as control. The PSH was calculated as follows:

$$\text{PSH}=\frac{A_{595}\text{control}-A_{595}\text{Sample}}{A_{595}\text{control}}$$

### 2.10 *In vitro* protein digestibility and free amino acids analysis

*In vitro* protein digestibility (IVPD) was carried out according to the reported method of Maliwal (Maliwal, 1983) with slight modification. Firstly, 0.5 g of the BMOP sample was added to 10 ml of deionized water, which pH was adjusted to 2.0 using 0.1 M HCl. 0.005 g pepsin was added and stirred for 2 hr under the temperature of 37°C to simulate the digestion of stomach. Secondly, the pH was adjusted to 7.0 using 0.1 M NaOH and 0.01 g trypsin was added. The mixture was stirred for 4 hr under 37°C to simulate the digestion of small bowel. After that, trichloroacetic acid was added into the whole system, reaching a final concentration of 10% (w/v). The supernatant was obtained by centrifugation at 4,000 × *g* for 10 min, in which the protein content was measured using Kjeldahl method. The IVPD was calculated as follows:

$$\text{IVPD}\left(\%\right)=\frac{{\text{Protein}}_{\mathrm{supernatent}}-{\text{Protein}}_{\mathrm{pepsin}}-{\text{Protein}}_{\mathrm{trypsin}}}{{\text{Protein}}_{\mathrm{sample}}}\times 100\%$$

After *in vitro* digestion, 5 ml of the liquid sample was vortexed evenly with 25 ml of cold acetone and centrifuged at 10,000 × *g* for 15 min. Three milliliters of the supernatant was derivatized with 2,4‐dinitro‐fluorobenzene (DNFB), and free amino acid was determined by the method of Elite‐AAK amino acid analysis system (Elite Analytical Instruments Co., Ltd., Dalian, China). The measurement conditions were as follows: Elite‐AKK amino acid analysis column (250 mm × 4.6 mm, 5 μm), column temperature at 27°C, 10 μl of injection volume, 1.2 ml/min of total flow of mobile phase, and the detection wavelength of 360 nm.

### 2.11 Statistical analysis

In the course of this study, all the experiments were performed in triplicate. Differences were considered statistically significant at *p* < 0.05. Results were evaluated using one‐way ANOVA using SPSS software (SPSS 18.0, SPSS Inc., Chicago, IL, USA) and processed by Origin software (Origin 8.5.1; OriginLab Corporation, Northampton, MA, USA).

## 3 RESULTS AND DISCUSSION

### 3.1 Morphology properties and particle size distribution

**Figure 1:**
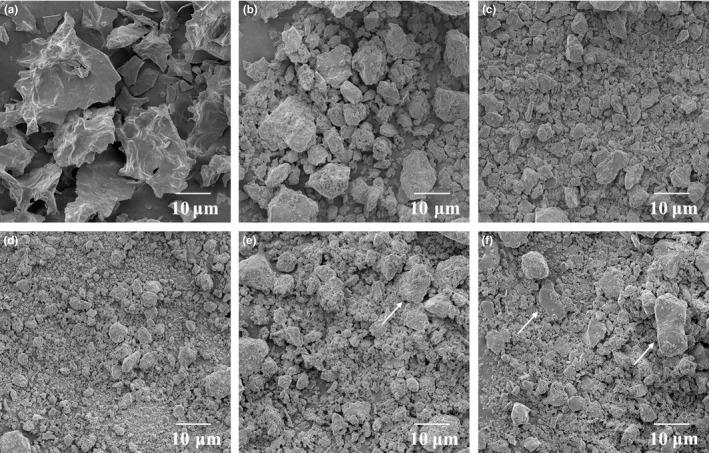
SEM for ball‐milled oyster protein (500 ×  magnification). (a) 0 min, (b) 4 min, (c) 8 min, (d) 12 min, (e) 16 min, (f) 20 min

In order to characterize the effect of ball milling treatment on morphology properties, SEM was firstly investigated, and the results are shown in Figure 1. The average diameter of protein particles was reduced to less than 20 μm (Figure 1b–f), which means that the ball milling pulverization carried out in this study could achieve the extent of superfine grinding. After ball milling treatment, the size of oyster protein powder significantly decreased and the powder became denser and more homogeneous in the first 12 min, which was similar to the effect on the ball‐milled soybean protein isolate and mussel protein (Sun, Wu, et al., 2015; Yu et al., 2018). Previously reports showed spherical protein particles after ball milling treatment, while the nonball‐milled particles were irregular (Li et al., 2017). However, with the lengthening of ball milling time, the diameter of the protein particles (arrows shown in Figure 1e,f) increased. The possible reason of this phenomenon was that, as a kind of soft material, the oyster protein particles were deformed during the excessive pulverization. The results indicated that the morphology of oyster protein powders could be significantly changed with high collision, shear force, and friction in the ball milling treatment. However, as a kind of soft material, the time of ball milling treatment should be paid attention to in the process of protein pulverization.

**Figure 2:**
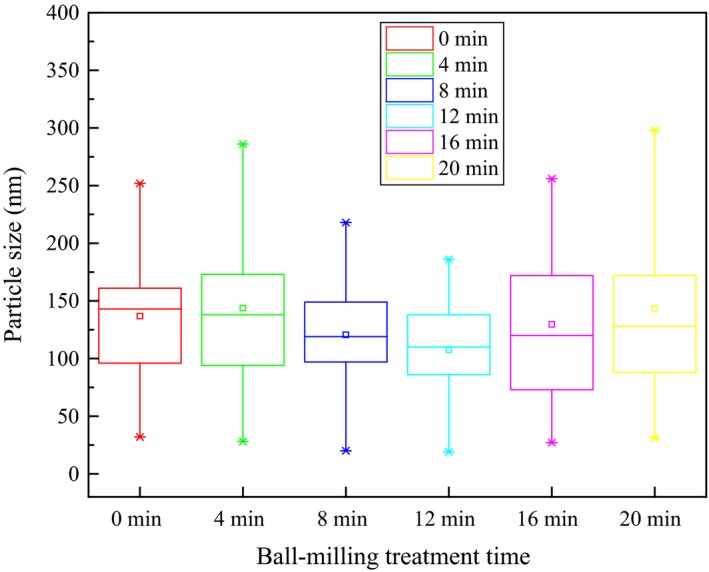
Particle size distribution of ball‐milled oyster protein

Particle size distribution of BMOP was determined, and the results were shown in Figure 2. After 12‐min ball milling treatment, the median of the particle size moved from 145 to 110 nm, which indicated that the particle size of BMOP decreased with the prolongation of ball milling time. However, after ball milling treatment for 16 and 20 min, the median of the particle size moved from 110 to 125 nm indicating that the particle size grew larger when the oyster protein was treated by excessive ball milling, resulting in a turning point at 12 min. It was also shown in the box chart that the particle size became uniform after 12‐min ball milling treatment. However, with the increase in ball milling time, the particle size distribution became dispersed. Other report showed that when the oyster protein was excessive ball‐milled, the surface force of protein particles were exposed, like water‐binding sites with surrounding water, which resulted in the particle agglomerate (Fadda, Cincotti, Concas, Pisu, & Cao, 2009).

### 3.2 Effects of ball milling on conformational structure

**Figure 3:**
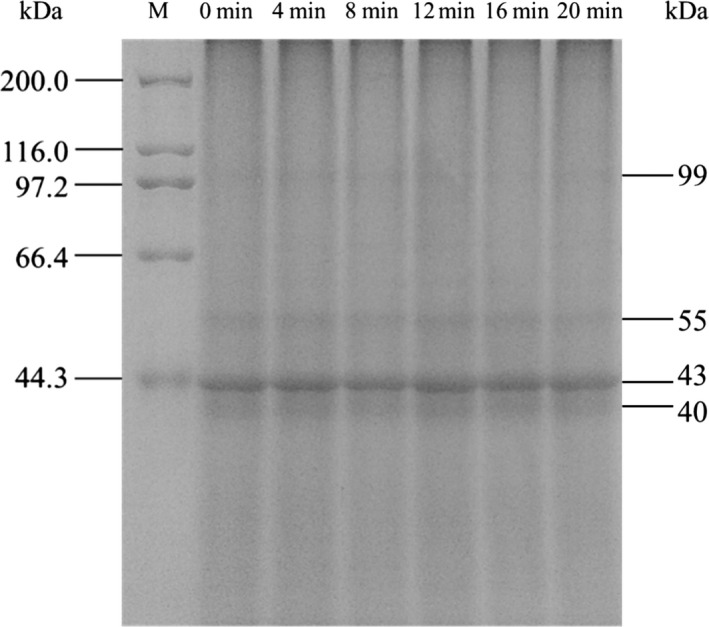
SDS‐PAGE of ball‐milled oyster protein. M: molecular weight markers

The conformational structure of BMOP was analyzed, SDS‐PAGE for primary structure analysis and CD for secondary structure. As the results shown in Figure 3, the bands of different ball milling time showed no obvious change in the molecular weight and protein concentration, which means that the primary structure of oyster protein did not altered after ball milling treatment. Similar results were known for whey protein and soybean protein isolate (Liu et al., 2017; Sun, Wu, et al., 2015). In addition, the electrophoresis bands are distributing at 99, 55, 43, and 40 kDa. It was speculated that the band of 99 kDa may be paramyosin (Woods, 1969) and the band of 43 kDa is likely to be actin (Wang, Wu, Jian, & Lu, 2008) according to findings in oyster.

The changes in secondary structure of BMOP were measured using far‐UV CD. As the data shown in Table 1, the major secondary structures of oyster protein were β‐sheet and random coil, which was similar to mussel protein (Yu et al., 2018). With the prolongation of ball milling time, no obvious changes were observed in the secondary structure of oyster protein. However, the minimum value of β‐turn 4.00% and the maximum value of β‐sheet 51.50% were observed in the oyster protein ball milling treated for 12 min, which indicated a relationship between the secondary structure and the changes of particle size.

**Table 1:** Effect of mall‐milling treatment on secondary structure of oyster protein

Ball milling time (min)	β‐Sheet (%)	β‐Turn (%)	Random coil (%)	α‐Helix (%)
0	48.60 ± 0.71^b^	5.00 ± 0.30^b^	46.10 ± 0.75^ab^	0 ± 0
4	48.73 ± 1.79^ab^	5.10 ± 0.10^b^	47.10 ± 1.65^bc^	0 ± 0
8	47.07 ± 0.70^a^	6.63 ± 0.25^c^	46.33 ± 0.59^b^	0 ± 0
12	51.50 ± 1.41^c^	4.00 ± 0.17^a^	44.60 ± 1.41^a^	0 ± 0
16	49.07 ± 1.00^b^	4.87 ± 0.29^b^	46.00 ± 1.23^ab^	0 ± 0
20	50.13 ± 1.55^bc^	5.23 ± 0.81^b^	44.63 ± 0.74^a^	0 ± 0

Different superscripted letter in the same column means significant differences (*P* < 0.05).

### 3.3 Effect of ball milling treatment on disulfide bond

**Figure 4:**
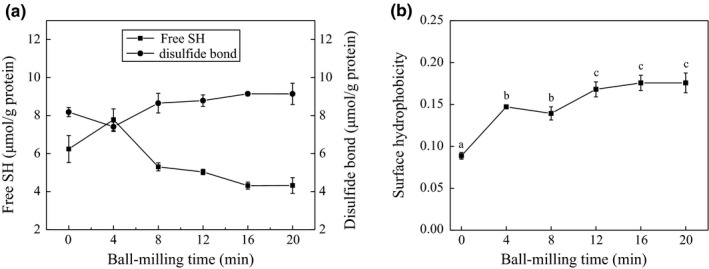
Changes of the content of disulfide bond (a) and surface hydrophobicity index (b) of ball‐milled oyster protein

As shown in previous research, content of disulfide bond and surface hydrophobicity index could partly reveal the tertiary and quaternary structure of protein (van Koningsveld et al., 2002). The results in Figure 4a showed the effects of ball milling treatment on the content of disulfide bond of oyster protein. The free SH increased to 7.78 μmol/g·protein firstly within ball milling treatment for 4 min, and then reduced gradually to 4.32 μmol/g·protein with the prolongation of ball milling time. Previous research showed that the changes of free SH were mainly due to the disruption or the generation of disulfide bond (Condés, Speroni, Mauri, & Añón, 2012; Jeyarajah & Allen, 1994; Sriket, Benjakul, Visessanguan, & Kijroongrojana, 2007). As a result, the content of disulfide bond showed an opposite trend, decreasing to 7.41 μmol/g·protein within 4 min followed by gradual increase. The ball milling treatment exposed more buried SH groups, which were eventually oxidized to form a disulfide bond during ball milling treatment (Sun, Liu, et al., 2015). As disulfide bond played an important role in the maintenance of the higher structure of protein, these results indicated that ball milling treatment with high collision, shear force, and friction enhanced the generation of disulfide bond resulting in an increase in the connection and aggregation of protein molecules.

### 3.4 Effect of ball milling on surface hydrophobicity index

Protein surface hydrophobicity index is closely associated with the higher structure of protein. It is also related to the functional properties of the protein (Kato & Nakai, 1980; Nakai & Li‐Chan, 1985). Thus, the effect of ball milling treatment on PSH of the oyster protein was investigated, and the results are shown in Figure 4b. Ball milling treatment increased the PSH significantly from 0.088 to 0.176. The increase in PSH indicated the conformational structure of protein molecules changed, exposing hydrophobic groups buried within the protein molecules. The transformation in the conformation of protein molecules resulted in the random aggregation of protein molecules and larger particle size. Similar results were reported in the ball‐milled whey protein and soybean protein isolate (Liu et al., 2017; Sun, Liu, et al., 2015).

### 3.5 Effect of ball milling on water‐holding capacity and oil‐binding capacity

**Figure 5:**
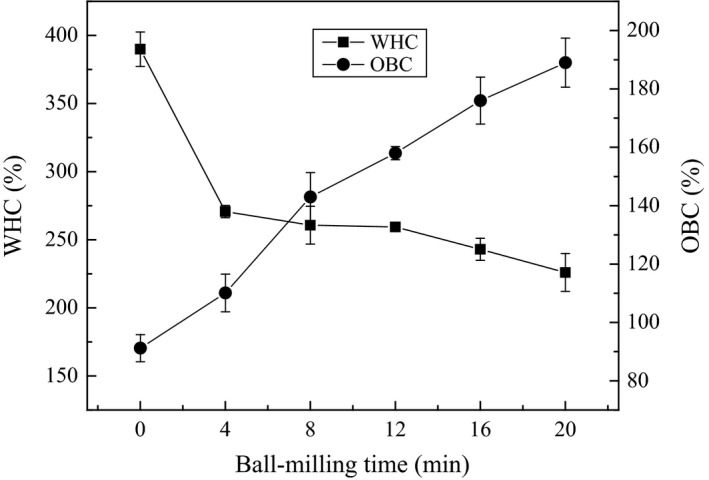
Effects of ball milling on water‐holding capacity and oil‐binding capacity of oyster protein

Alterations in conformational structure and particle size may cause the changes of functional properties. Therefore, the effect of ball milling on WHC and oil‐binding capacity (OBC) was measured. The WHC is a significant property of protein materials for industrial applications, which may be related to vapor adsorption or protein gel formation (Hu, Li‐Chan, Li, Tian, & Pan, 2013; Hu, Wu, Zhu, Zhang, & Xu, 2013). The WHC of BMOP shown in Figure 5 had a sharp decrease from 390% to 259% with increasing ball milling time from 0 to 4 min and a slow reduction from 259% to 226% when the ball milling time increased from 4 to 20 min. Meanwhile, the OBC of BMOP obviously increased from 91.2% to 189.1% after 20 min ball milling treatment. As WHC and OBC are related to the alterations of conformation, the protein molecules with increasing exposure of hydrophobic groups were easier to combine oil molecules rather than water molecules.

### 3.6 *In vitro* protein digestibility and free amino acid analysis

**Figure 6:**
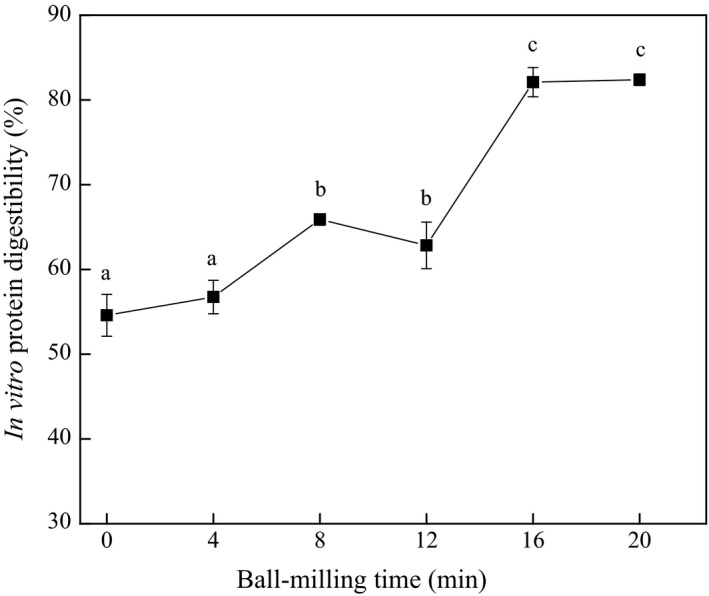
Effect of ball milling on *in vitro* protein digestibility

Not only the physicochemical properties, the changes in the conformation structure and particle size but also affect the digestibility. As shown in Figure 6, the *in vitro* protein digestibility significantly increased from 54.6% to 82.4% with the prolongation of ball milling time. It was reported that the decrease in diameter of protein particles could enhance the digestibility (Tinus, Damour, Van, & Sopade, 2012). Therefore, the decrease in diameter of protein particles was the main reasons for the increase in oyster protein digestibility within 12‐min ball milling treatment. However, when ball milling treated for more than 16 min, the protein digestibility increased even higher, while the particle size of oyster protein grew larger observed by SEM. This may be due to the protein denaturation caused by ball milling treatment, which has been confirmed in the analysis of the digestibility of hammer‐milled peas (Nguyen, Gidley, & Sopade, 2015).

The free amino acids after *in vitro* protein digestion were determined, and the results are shown in Table 2. It was shown that BMOP was abundant in eight essential amino acids for human body, while the Arg, Leu, Trp, Lys, and Tyr were predominant amino acids in the oyster protein. The results were similar to the protein hydrolysate of oyster (*Crassostrea talienwhanensis*). The maximum content total amino acid was found in the protein sample without ball milling treatment which seemed unreasonable, as ball milling treatment resulted in a better digestibility. This may be caused by the differences in the extent of protein hydrolysis. When the oyster protein was hydrolyzed to oligopeptides of 2–5 aa, the protein was also considered to be digested, while the amount of free amino acids did not increased. As many peptides containing 2–5 aa were reported during digestion (Umayaparvathi et al., 2014), the digestion of BMOP may produce more functional and bioactive peptides, which need to be further studied in the future.

**Table 2:** Amino acid analysis of ball‐milled oyster protein digestion (mg/g·protein)

Amino acid	Ball milling treatment (min)					
	0	4	8	12	16	20
Asp	1.023 ± 0.027^ab^	0.932 ± 0.085^a^	0.857 ± 0.017^a^	1.170 ± 0.107^c^	1.055 ± 0.015^bc^	0.887 ± 0.002^a^
Glu	1.921 ± 0.156^b^	1.427 ± 0.078^a^	1.332.±0.038^a^	1.826 ± 0.039^b^	1.743 ± 0.052^b^	1.680 ± 0.001^b^
Ser	2.251 ± 0.125^c^	1.967 ± 0.148^ab^	1.679 ± 0.051^a^	2.621 ± 0.138^d^	1.895 ± 0.016^b^	1.662 ± 0.005^ab^
Arg	18.930 ± 1.651^c^	15.166 ± 1.319^a^	14.626 ± 0.942^a^	17.416 ± 0.716^bc^	14.460 ± 1.001^a^	15.671 ± 0.018^ab^
Gly	0.434 ± 0.005^ab^	0.512 ± 0.029^bc^	0.525 ± 0.062^c^	0.909 ± 0.053^e^	0.784 ± 0.059^d^	0.378 ± 0.000^a^
Thr	1.928 ± 0.038^d^	1.315 ± 0.043^b^	1.123 ± 0.010^a^	2.156 ± 0.112^e^	1.863 ± 0.014^d^	1.676 ± 0.002^c^
Pro	0.434 ± 0.007^ab^	0.428 ± 0.044^a^	0.495 ± 0.019^b^	0.778 ± 0.099^c^	0.696 ± 0.038^c^	0.389 ± 0.000^a^
Ala	1.779 ± 0.060^b^	1.614 ± 0.030^a^	1.453 ± 0.090^a^	2.063 ± 0.061^c^	1.835 ± 0.103^b^	1.528 ± 0.000^a^
Val	2.725 ± 0.092^d^	2.253 ± 0.020^b^	1.839 ± 0.018^a^	2.677 ± 0.092^d^	2.385 ± 0.067^c^	2.275 ± 0.002^c^
Met	2.042 ± 0.162^cd^	1.664 ± 0.093^a^	1.580 ± 0.054^a^	2.064 ± 0.095^d^	1.822 ± 0.027^bc^	1.679 ± 0.002^ab^
Cys	0.617 ± 0.032^bc^	0.563 ± 0.010^ab^	0.516 ± 0.032^a^	0.681 ± 0.084^c^	0.540 ± 0.019^ab^	0.617 ± 0.001^c^
Ile	2.505 ± 0.050^b^	2.495 ± 0.092^b^	2.173 ± 0.092^a^	2.920 ± 0.156^c^	2.494 ± 0.019^b^	2.296 ± 0.003^b^
Leu	12.223 ± 1.036^c^	9.510 ± 0.0.364^a^	8.471 ± 0.591^a^	10.552 ± 0.188^b^	8.994 ± 0.285^a^	10.389 ± 0.009^b^
Trp	14.999 ± 1.175^d^	12.636 ± 1.113^c^	8.967 ± 0.377^a^	12.186 ± 0.267^c^	10.596 ± 0.660^b^	11.791 ± 0.017^c^
Phe	3.221 ± 0.225^d^	2.720 ± 0.271^c^	2.503 ± 0.242^c^	1.300 ± 0.065^b^	1.373 ± 0.053^b^	0.854 ± 0.001^a^
His	0.753 ± 0.015^b^	0.689 ± 0.005^ab^	0.617 ± 0.014^a^	2.730 ± 0.074^c^	3.208 ± 0.055^d^	3.829 ± 0.003^e^
Lys	14.290 ± 1.200^c^	11.556 ± 0.952^ab^	11.586 ± 0.695^ab^	12.366 ± 0.228^c^	10.716 ± 0.640^a^	11.504 ± 0.010^ab^
Tyr	11.069 ± 0.567^c^	8.955 ± 0.705^ab^	8.256 ± 0.632^ab^	8.517 ± 0.140^ab^	7.614 ± 0.659^a^	8.697 ± 0.010^b^
Total	93.142 ± 6.327^d^	76.401 ± 5.401^a^	65.899 ± 3.977^b^	84.933 ± 2.712 ^cd^	74.073 ± 3.779a^b^	77.803 ± 0.087^bc^

Different superscripted letter in the same row means significant differences (*P* < 0.05).

## 4 CONCLUSIONS

Ball milling treatment significantly reduced the particle size of oyster protein, and also changed the molecular conformation and the surface hydrophobicity index of oyster protein molecules. The combining of these changes affected the water‐holding capacity, oil‐binding capacity, and *in vitro* digestibility of BMOP. Proper processing of ball milling treatment appears to be a very effective process to produce oyster protein powders with smaller particle size, better oil‐binding capacity, and digestion for peptides. This study provided a theoretical basis for the application of ball milling in the processing of oyster protein powder.

## CONFLICT OF INTEREST

There is no conflict of interest.
